# Novel Applications in Controlled Drug Delivery Systems by Integrating Osmotic Pumps and Magnetic Nanoparticles

**DOI:** 10.3390/s24217042

**Published:** 2024-10-31

**Authors:** David Navarro-Tumar, Belén García-Merino, Cristina González-Fernández, Inmaculada Ortiz, Ma.-Fresnedo San-Román, Eugenio Bringas

**Affiliations:** Department of Chemical and Biomolecular Engineering, ETSIIT, University of Cantabria, Avda. de los Castros, s/n, 39005 Santander, Spain; david.navarro@unican.es (D.N.-T.); belen.garcia@unican.es (B.G.-M.); cristina.gonzalezfdez@unican.es (C.G.-F.); inmaculada.ortiz@unican.es (I.O.); maria.sanroman@unican.es (M.-F.S.-R.)

**Keywords:** osmotic pumps, drug delivery, magnetic nanoparticles, forward osmosis, medical devices, draw solutions, osmotic pressure

## Abstract

The alarming rise in chronic diseases worldwide highlights the urgent need to overcome the limitations of conventional drug delivery systems. In this context, osmotic pumps are able to release drugs by differential osmotic pressure, achieving a controlled rate independent of physiological factors and reducing the dosing frequency. As osmotic pumps are based on the phenomenon of osmosis, the choice of high osmolality draw solutions (DSs) is a critical factor in the successful delivery of the target drug. Therefore, one alternative that has received particular attention is the formulation of DSs with magnetic nanoparticles (MNPs) due to their easy recovery, negligible reverse solute flux (RSF), and their possible tailor-made functionalization to generate high osmotic gradients. In this work, the possible integration of DSs formulated with MNPs in controlled drug delivery systems is discussed for the first time. In particular, the main potential advantages that these novel medical devices could offer, including improved scalability, regeneration, reliability, and enhanced drug delivery performance, are provided and discussed. Thus, the results of this review may demonstrate the potential of MNPs as osmotic agents, which could be useful for advancing the design of osmotic pump-based drug delivery systems.

## 1. Introduction

Noncommunicable or chronic diseases (NCDs) are long-term conditions caused by a variety of genetic, physiological, environmental, and behavioural factors. These include cardio-vascular and chronic respiratory diseases, cancer, and diabetes [1]. It is estimated that deaths from NCDs have reached 40 million each year, accounting for 74% of all deaths worldwide [1,2]. These are mostly in low-income countries. Without immediate action, NCD-related deaths are expected to reach 86% of the projected 90 million deaths per year by 2050 [3]. The global health crisis of the COVID-19 pandemic has highlighted the urgent need to improve access to NCD treatments [2]. In the context of this health crisis, a significant proportion of the population faced challenges in accessing essential medicines for the treatment of NCDs, resulting in treatment interruptions and serious health consequences for patients [4]. In accordance with Target 3.4 of Sustainable Development Goal 3 “Health and well-being” of the 2030 Agenda, which states that “By 2030, reduce premature mortality from noncommunicable diseases by one-third through prevention and treatment, and promote mental health and well-being” [5], there is an urgent need for concerted efforts to develop more effective and affordable treatments to reduce mortality. One potential strategy to address the increasing number of deaths associated with NCDs is the development of novel medical technologies and robust devices to make necessary treatments cost-effective and feasible with a minimal drug administration frequency, and to ensure easier access and adherence to treatment, particularly for patients in the most vulnerable settings [6,7,8].

In this context, conventional drug delivery systems are unable to effectively regulate the release of drugs at the target site [9]. This results in suboptimal drug concentrations in the plasma, which can lead to treatment failure and the potential for adverse effects [10]. In contrast, controlled drug delivery systems, such as osmotic pumps, have been reported to fit to zero-order kinetics with independence of external factors, such as the gastric pH, food, and hydrodynamic conditions [11]. These maintain adequate plasma concentrations and enhance patient adherence to treatment by minimizing the dosing frequency [12]. Consequently, controlled drug delivery systems overcome the limitations of conventional drug delivery methods and have significant potential to improve the treatment of NCDs.

Drug delivery systems based on osmotic pumps are based on the osmosis phenomenon, known as forward osmosis (FO), where the release of drugs is triggered by a trans-membranal pressure difference between two fluids, a feed solution (FS), and a high osmolality draw solution (DS) [13,14]. FO is considered a promising membrane process due to its simple operation, low membrane fouling, and low energy consumption, as it is based on the difference in chemical potential across both sides of the membrane. FO has primarily been applied to seawater desalination, sewage reclamation, and industrial wastewater treatment, addressing the pressing issue of freshwater scarcity [15,16,17]. However, this technology also has potential for various applications, such as food processing, power generation [18], and drug delivery systems [19]. One significant drawback of FO is the energy cost associated with the DS regeneration stage. This makes the overall process unfeasible [13,20]. To overcome this obstacle, the use of magnetic nanoparticles (MNPs) to formulate the DS has received special interest since their superparamagnetic properties can be induced by an external magnetic field, significantly reducing the cost of the regeneration step [13,14]. The advantages of their superparamagnetic properties have been leveraged in several biomedical applications, including targeted drug delivery and the extraction of biomolecules [21,22,23]. Additionally, MNPs have been used in sample preparation as a preconcentration method for biomarkers of interest from biological fluids, enhancing the analytical signal and eliminating potential interferences [23,24]. In this regard, FO can also be used as continuous preconcentration method for biologically relevant biomolecules before sensing, bringing the analytes into the detection range of the sensors and improving detection, regardless of the sensing modality [25]. The reported benefits of MNPs and osmotic pumps lead to their integration in the development of novel drug delivery systems with excellent performance. Regarding this issue, MNPs have already been integrated into osmotic pumps in previous studies. Zaher et al., in 2015, integrated magnetic nanocomposite membranes composed of MNPs and thermoresponsive polymers such as poly(N-isopropylacrylamide) into osmotic pumps for drug delivery. These membranes can be magnetically triggered by an external magnetic field, functioning as a switchable on-demand valve. The magnetic triggering increases the porosity of the membrane, resulting in a desired change in the drug release profile [26].

Despite the considerable potential offered by integrating MNPs and osmotic pumps, to the best of our knowledge, there are still no studies that have explored the use of MNPs as DSs in osmotic pumps for the development of novel medical devices for drug delivery. The objective of this review is to establish the foundation and propose, for the first time in the literature, an approach for the creation of novel drug delivery devices based on osmotic pumps. This combination could offer a number of attractive advantages, including controlled drug delivery systems with higher reusability and the elimination of the need for batteries. The precision of drug release rates in osmotic pumps can be enhanced by the integration of MNPs, which maintain the osmotic pressure difference due to their low RSF. Additionally, the possibility of delivering more than one drug simultaneously and the inclusion of stimuli-responsive materials, such as superparamagnetic materials, would contribute to the advancement of even more personalized treatments for patients. The potential advantages of the proposed systems could contribute to the development of novel, effective, and cheaper drug delivery devices and more personalized treatments for NCDs, improving healthcare worldwide. This would have significant impacts in low-income countries by facilitating access to continuous and specific treatments for NCDs through these systems. Therefore, this study represents a significant advancement of the state-of-the-art and contributes to the continuous improvement of currently available medical devices in order to address the current global health challenges.

## 2. Osmotic Pumps

An osmotic pump for drug delivery is based on a system that is capable of releasing a drug from a reservoir at a constant rate. This is achieved by means of the osmotic pressure difference between the osmotic agent and the interstitial fluids of the patient [19]. The osmotic pump is composed of a core that stores the osmotic agent and the drug, whether in liquid or solid state, surrounded by an external semipermeable membrane with an orifice for its release. Consequently, the water flux into the device increases the volume of the osmotic agent reservoir, thereby releasing the drug through the delivery office [27]. In certain instances, if the drug is highly soluble in water, the utilization of an additional osmotic agent may be unnecessary, as the drug itself is capable of generating sufficient osmotic pressure for its delivery [11]. Consequently, the delivery of drugs in osmotic pumps is independent of physiological factors, but is influenced by a number of factors, including the solubility of the drug, the size of the delivery orifice, the osmotic pressure in the core, and the type, surface area, and thickness of the semipermeable membrane [9,27].

**Figure 1:**
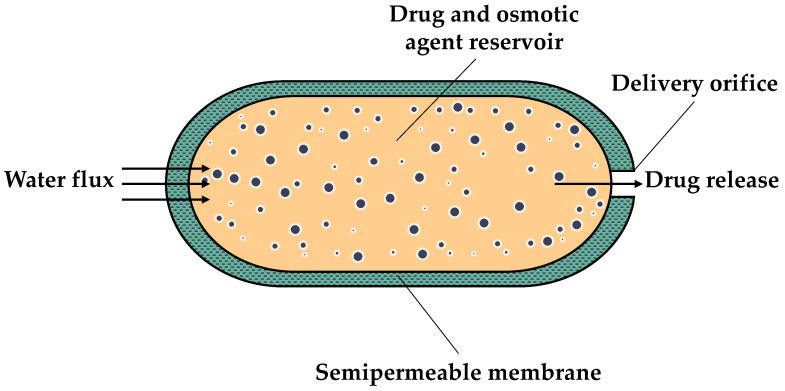
Schematic diagram of an elementary osmotic pump (EOP).

These drug delivery systems can be employed for both oral treatment and subcutaneous implantation, encompassing a range of systems, including elementary osmotic pumps (EOPs), push–pull osmotic pumps (PPOPs), controlled porosity osmotic pumps (CPOPs)**,** Rose–Nelson pumps (RNPs), Higuchi–Leeper pumps (HLPs), and Higuchi–Theeuwes pumps (HTPs) [9]. For further details about osmotic pumps, including information about their composition or details about other types of osmotic pumps, such as the RNP, HLP or HTP, readers are directed to the excellent reviews published by Patel et al. in 2021 and by Almoshari in 2022 [28,29]. Figure 1 presents a schematic diagram of an EOP.

In recent years, studies have been conducted with the objective of improving oral osmotic pumps, with a particular focus on the EOPs, PPOPs, and CPOPs. In particular, the EOPs represent the simplest and earliest form of osmotic pump used in humans [11,30]. This drug delivery system represents a simplified version of the HTP, which was originally developed for the controlled release of water-soluble drugs [30,31]. As illustrated in Figure 1, an EOP comprises a core containing the drug with an osmotic agent, or without it if the drug itself is capable of generating sufficient osmotic pressure for its delivery. The aforementioned core is then covered by a semipermeable membrane, typically cellulose acetate, which is subsequently drilled with a delivery orifice [11,30]. Thus, when the osmotic pump comes in contact with an aqueous environment, the osmotic pressure difference generated by the osmotic agent causes water to pass through the semipermeable membrane, thus increasing the volume inside the EOP. Consequently, this leads to an increase in hydrostatic pressure inside the EOP, resulting in the release of the drug through the delivery orifice [30]. Despite its advantages, there are aspects of EOPs that require improvement. These include the complexity of the preparation methods, the use of drilling and laser techniques to prepare the delivery orifice, and the enlargement of the delivery orifice during drug release [32].

**Figure 2:**
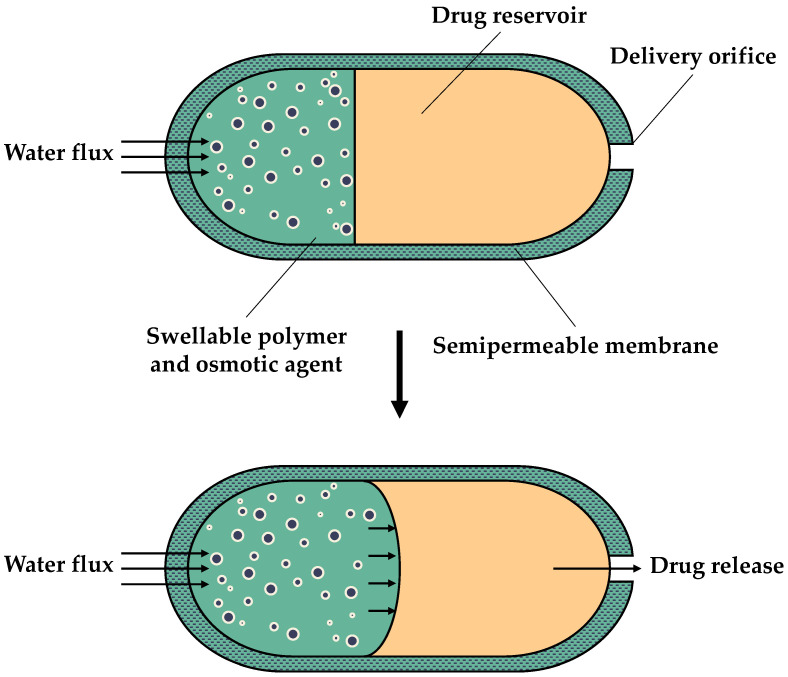
Schematic diagram of a push–pull osmotic pump (PPOP).

PPOPs were initially developed for the delivery of insoluble drugs [31]. As depicted in Figure 2, PPOPs comprise two compartments: a push layer, comprising a swellable polymer with an osmotic agent, and an active drug compartment with a delivery orifice. The entire core is covered by a semipermeable membrane. During use, water enters both compartments, resulting in the expansion of the polymer layer and pumping of the drug solution, either dissolved or in suspension, outside the osmotic pump through the delivery orifice [10,33]. This type of osmotic pump has a higher cost compared to other types of extended drug release systems [33].

**Figure 3:**
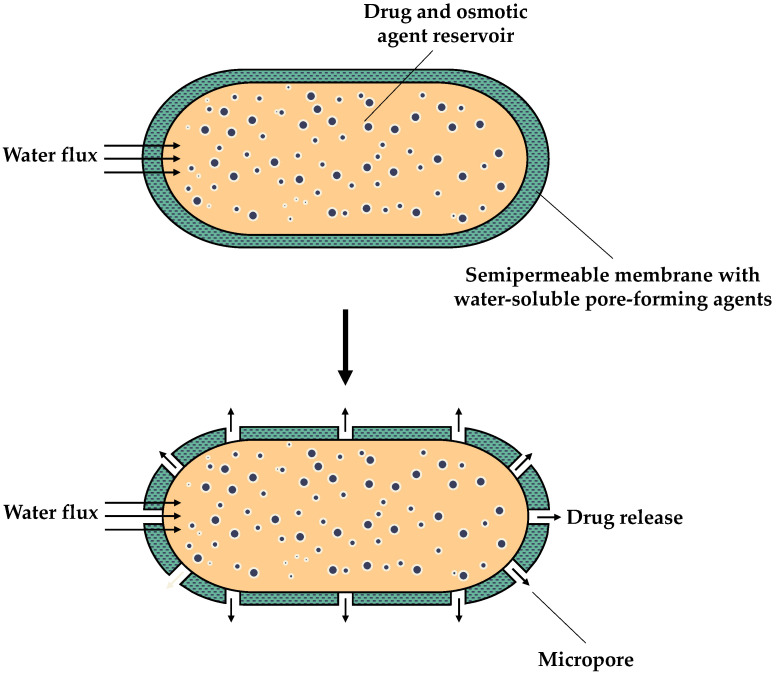
Schematic diagram of a controlled porosity osmotic pump (CPOP).

Finally, as illustrated in Figure 3, a CPOP is comprised of a core containing the drug and the osmotic agent. The aforementioned core is then covered by a semipermeable membrane with an asymmetric structure, as described in detail in reference Akhtar et al. in 2022 [9]. As opposed to other osmotic pumps, CPOPs do not have a delivery orifice for drug release. In contrast, the membrane contains water-soluble pore-forming agents [9]. Upon contact with water, the pore-forming agents present in the membrane undergo dissolution, resulting in the formation of pores. Consequently, the drug is released through the microporous membrane [34], either in solution or suspension [32]. Therefore, the mechanism of drug release is contingent upon the hydrostatic pressure and the formation of pores in the membrane. Furthermore, CPOPs offer a distinct advantage since they do not require laser drilling, which simplifies the manufacturing process [34].

**Figure 4:**
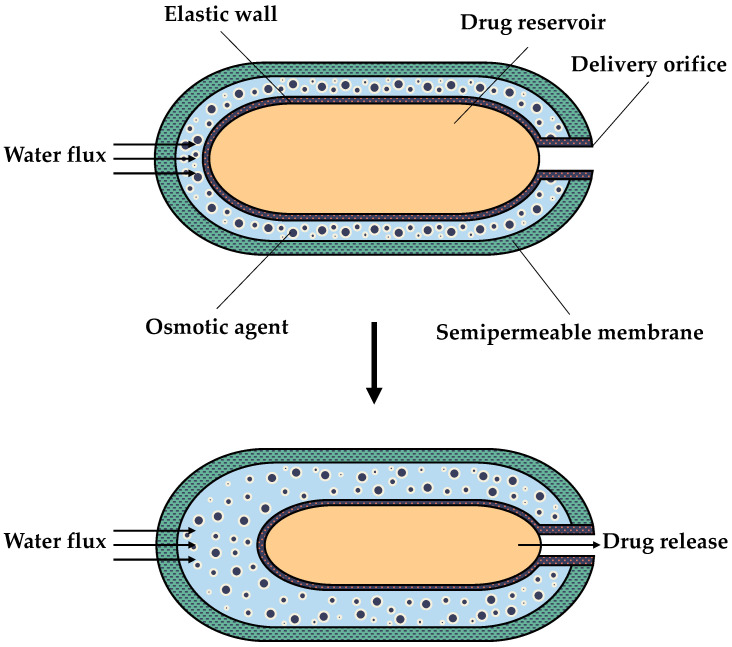
Schematic diagram of a Higuchi–Theeuwes pump (HTP) [37].

Commercial osmotic pumps, such as ALZET^®^ (Durect Corporation, Cupertino, CA, USA), are based on the HTP [35], as shown in Figure 4. They are the most commonly used osmotic pumps for in vivo preclinical therapeutic research involving animals. Once implanted subcutaneously in the animal, these pumps ensure a constant rate of delivery of the investigated drug for a maximum period of 28 days. This method of drug administration presents several advantages for research purposes over conventional delivery systems, including (i) the maintenance of a constant concentration of the drug to maximize its efficacy and reduce adverse effects, (ii) the elimination of the need for researcher intervention during the experiment, and (iii) the time savings by removing the need for frequent handling and repetitive injection of the animal [36]. Although implanted osmotic pumps require general anaesthesia for administration and removal, this is typically manageable in wildlife rehabilitation centres and zoos, where anaesthesia is commonly utilised for most procedures [19]. Consequently, the ALZET^®^ device enables the assessment of long-term drug efficacy during the investigative phase [36].

The potential of osmotic pumps as a drug delivery device is obvious, as evidenced by the numerous commercial treatments based on this technology listed in Table 1. On the other hand, osmotic drug delivery systems based on osmotic pumps, including CPOPs [9,34], PPOPs [10], and EOPs [11,12], whether implantable or orally administered devices, are constantly being developed by medical researchers. These include the treatment of hearing loss [38], multiple sclerosis [39], hypertension [40], and Duchenne muscular dystrophy [41]. Table 2 shows the most recent studies on osmotic pumps, grouped by route of administration: (i) oral, (ii) implanted, or (iii) in vitro tests. 

It is therefore worth highlighting the significant importance of osmotic drug delivery devices, not only as a widely used tool in clinical research but also because of the existence of commercial treatments based on this type of systems. This fact underlines the feasibility and advantages of osmotic pumps in the field of controlled drug release. In this context, an understanding of the phenomena underlying osmotic pump-based drug delivery systems is crucial to progress in the design of these devices. Therefore, a brief description of the FO phenomenon is given in the next section.

## 3. Osmotic Pump Operation: The Forward Osmosis Phenomenon

**Figure 5:**
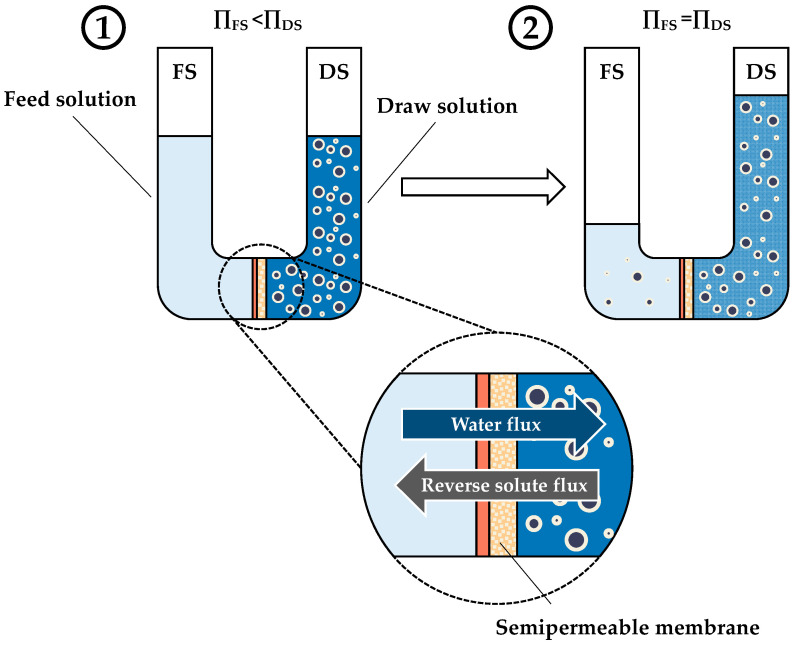
Scheme of the FO process: (**1**) FO process at the initial stage and (**2**) FO process at equilibrium.

As illustrated in Figure 5, forward osmosis (FO) is a natural process whereby water flux is generated by the osmotic pressure gradient (Δ*π*) between a low osmotic pressure solution, designated as a feed solution (FS), and a high osmotic pressure solution, designated as a draw solution (DS), separated by a semipermeable membrane [13,17]. During the process, water molecules migrate from the FS to the DS, thereby diluting the DS and reducing the osmotic pressure difference between the two phases. Once the equilibrium between the two phases has been reached, the process remains stable [56]. 

The water flux in FO processes is determined by the following equation [17]:

(1) $$J_{w}=A(\pi_{DS}-\pi_{FS})$$

where $J_{w}\left(m\;s^{-1}\right)$ is the water flux, $A\left(m\;Pa^{-1}\;s^{-1}\right)$ is the membrane permeability, and $\pi_{DS}$ and $\pi_{FS}\;(Pa)$ are the bulk osmotic pressure of the DS and FS, respectively. 

The solute flow across the membrane is described by Fick’s law, as given in Equation (2) [57]:

(2) $$J_{s}=B∆C$$

where $J_{s}\;\left(kg\;m^{-2}\;s^{-1}\right)$ is the solute flux, $B\left(m\;s^{-1}\right)$ is the solute permeability within the membrane, and $∆C\left(kg\;m^{-3}\right)$ is the concentration gradient of solute across the active layer of the membrane. 

The selection of the most appropriate DS and its regeneration stage has a significant impact on the FO process. Therefore, for general applications the DS must (i) create a significant osmotic pressure difference between the FS and DS, (ii) have a negligible reverse solute flux (RSF), (iii) be available in sufficient quantities, (iv) be affordable, (v) be non-toxic, and (vi) be easy and inexpensive to regenerate after the process [56]. Thus, DS options include inorganic and organic salts, volatile compounds, polyelectrolyte and switchable polarity solutions [14], and others such as functionalized nanoparticles and Na^+^-functionalized carbon quantum dots [58], carefully selected in accordance with the specific application. Although commercial compounds such as glucose, sodium chloride, magnesium chloride, or magnesium sulphate have been used as DSs, these compounds cause reverse solute flux (RSF) and water contamination and require high-energy processes for regeneration, making them challenging for large-scale use [59]. On the other hand, the DS regeneration stage is crucial for the sustainability of the FO process. After the FO process, the osmotic gradient is reduced due to the dilution of the DS. As a result, the excess water in the DS can be extracted in order to recover the DS for future use [13]. Conventional DS recovery is based on energy-intensive membrane and thermal desalination processes, such as reverse osmosis (RO), ultrafiltration (UF), or membrane distillation (MD), which have negative impacts on the energy consumption of the overall process, thus affecting the viability of the FO process [13,20]. Consequently, the economic viability of the FO process would be improved if a sustainable, effective, and low-cost regeneration phase could be achieved [13].

## 4. Magnetic Nanoparticles as Draw Solutions in Forward Osmosis

In response to these issues, MNPs have received particular attention over traditional DSs because they ensure an efficient regeneration stage and negligible RSF. MNPs consist of nanoparticles (NPs) of pure iron, nickel, cobalt, and their oxides, ferrites, and metallic alloys. The main characteristic of these nanomaterials is their superparamagnetic behaviour, a phenomenon that appears below a certain particle diameter depending on the material. Superparamagnetic materials exhibit high magnetic saturation values that are several orders of magnitude higher than those typical of paramagnetic materials, resulting in a strong response to an external magnetic field [21,60,61,62], and the highest value of magnetization that a material can achieve is known as saturation magnetization. In addition, coercivity and remanent magnetization are important magnetic properties to consider when formulating DSs with MNPs. Coercivity indicates the required magnetic field to demagnetize the materials, while remanent magnetization indicates the magnetization of the materials in absence of an applied magnetic field. For superparamagnetic materials, both coercivity and remanent magnetization are equal to zero [63,64]. Hence, magnetic nanoparticles, due to their superparamagnetic behaviour, do not exhibit magnetic properties unless an external magnetic field is applied. Thus, their magnetic moment vectors relax in the absence of an external magnetic field, resulting in minimal attraction between particles, which reduces the risk of agglomeration [21,60,61,62,63]. Minimizing agglomeration also reduces the decrease in water flux obtained using the MNPs as the DS in several cycles of use [56]. These superparamagnetic properties also lead to a sensitive response when an external magnetic field is applied [63], which would facilitate the recovery of the MNPs for subsequent use as a DS. Therefore, the superparamagnetic properties of MNPs allow them to be easily recovered using an external magnetic field. 

**Figure 6:**
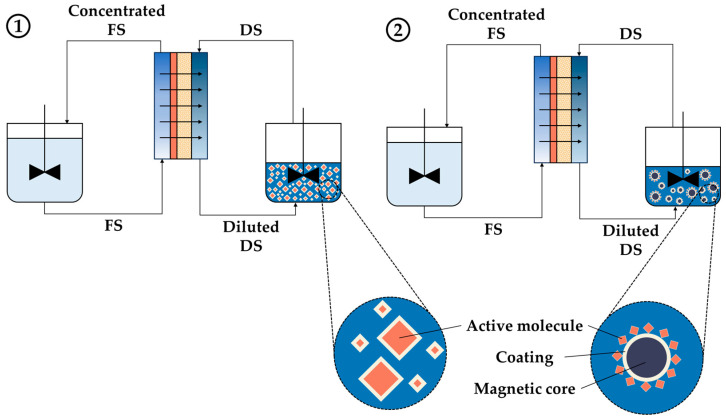
Scheme of the FO process: (**1**) FO process with a conventional DS and (**2**) FO process with MNPs as the DS.

However, bare MNPs are unable to generate sufficient osmotic pressure or disperse properly, leading to agglomeration and difficulty in their separation from the diluted DS, which limits their application as a DS [14,59]. These drawbacks can be overcome by a tailor-made coating or functionalization, as shown in Figure 6, which is facilitated by the easily modifiable surface of MNPs [56]. Smaller sizes of MNPs result in a higher coated/functionalized surface area/volume ratio, which is important to consider in order to generate high osmotic pressure as it affects the performance of the process [65]. For example, their osmotic pressure, hydrophilicity, and dispersibility can be efficiently increased by modifying the NP surface with low-molecular-weight and highly water-soluble polymers [59].

Therefore, the advantages of using coated/functionalized MNPs as the DS include (i) a high water flux, (ii) low energy, (iii) biocompatibility [56], (iv) high surface area/volume ratio, (v) low toxicity [66], (vi) easy recovery of the particles by a magnetic field due to their superparamagnetic behaviour, and (vii) the possibility of reuse [56,66]. 

A systematic review was conducted using Scopus as the database to identify relevant articles on the use of MNPs as the DS in FO. The search employed the following keywords: “draw solution”, “magnetic nanoparticle*”, and “forward osmosis” combined with the Boolean operator “and”. Only original research articles were considered, with no restriction on the publication date, resulting in the identification of 35 scientific articles on the topic. Thus, Table 3 presents the advances in the use of MNPs as osmotic agents in FO processes, differentiated by the compound used for coating/functionalization: (i) MNPs coated/functionalized with organic acids and their derivatives, (ii) MNPs coated/functionalized with organic polymers, (iii) MNPs coated/functionalized with polysaccharides, (iv) MNPs coated/functionalized with other organic compounds, and (v) bare MNPs and MNPs coated/functionalized with inorganic compounds. 

The literature review in Table 3 indicates that a large variety of compounds are used for coating/functionalizing MNPs, from organic compounds, such as tri-sodium citrate or polyethylene glycol, to inorganic compounds, such as EDTA or potassium. These can be classified into five categories: (i) organic acids and their derivatives, (ii) organic polymers, (iii) polysaccharides, (iv) other organic compounds, and (v) bare and inorganic compounds. Nevertheless, most of the studies reviewed in Table 3 employed organic compounds to coat and functionalize the MNPs. These organically coated MNPs have received particular attention due to the advantages they offer as DSs, including (i) improved stability [56], (ii) a high surface area/volume ratio, (iii) easy tuning of the osmotic properties by surface modification, (iv) easy recovery due to superparamagnetic properties, (v) non-toxicity, and (vi) a low RSF due to large molecular sizes [73,89]. Consequently, the aforementioned properties, including the osmotic pressure and RSF, are contingent upon the organic coating. While the organic coating is a crucial factor to consider when synthesizing coated/functionalized MNP for use as a sustainable DS, it may affect their magnetic properties, thereby impeding particle recovery [89]. In this context, the use of specific organic compounds stands out from others. Polyacrylic acid, polyethylene glycol, and poly-sodium acrylate are the most frequently used organic compounds. Nevertheless, it is not possible to establish a reliable correlation between the same functionalization compounds in MNPs due to differences in the experimental conditions of the synthesis and a lack of information in the studies.

Regarding the synthesis method, the most commonly employed for MNPs is co-precipitation, as shown in Table 3. This is a straightforward, cost-effective, and extensively researched alternative, independently of the compound used for its functionalization [61,62]. The exception to this is the case of MNPs synthesized by coating with polymers, where the thermal decomposition method is the most commonly employed. Yang et al. employed polyglycerol as a coating material for MNPs and carried out the functionalization via thermal decomposition [80,81,82]. 

With regard to the particle properties, most of the articles presented in Table 3 describe MNPs as having a size within the range of 3–40 nm. The differences in size among MNPs synthesized by the same method are highlighted, such as for tri-sodium citrate-, poly-sodium acrylate- or polyacrylic acid-coated MNPs, where size variations of an order of magnitude can be observed. It is of significant importance to note that the synthesis method plays a pivotal role in determining the properties of the particle, including the size. Particle size depends on a number of variables, including the residence time, reactant flow rates, precursor concentration, pH, and temperature. Even minor alterations to the synthesis method can have a profound impact on the particle properties, resulting in disparate particle sizes [61].

Once the MNPs have been synthesised, their potential as a DS in FO systems was evaluated. This was typically performed using membranes in the range 1.8–180 cm^2^ with a predominance of cellulose triacetate as the FO membrane material, as shown in Table 3. Generally, commercial membranes are used, highlighting suppliers such as Hydration Tech. Innovations, Porifera Inc. or Aquaporin A/S. The functionalization of MNPs enables the attainment of a sufficient osmotic pressure to concentrate the FS, typically using deionized water or an NaCl solution as the FS, resulting in water fluxes comparable to those obtained with a conventional DS but with a negligible RSF. For instance, polyacrylic acid-coated MNPs with a concentration of 0.08 M have been observed to achieve water fluxes of 12.0–13.9 LMH when using deionized water as the FS and 3.0–6.3 LMH when using a 3.5% (*w*/*w*) NaCl solution [70,71]. An interesting observation pertains to the utilization of MNPs coated with tri-sodium citrate. In both cases presented in Table 3, cellulose triacetate membranes from the supplier Hydration Tech. Innovations were employed. Concentrations of 0.02–2.00 g·L^−1^ of the tri-sodium citrate-coated MNPs resulted in fluxes of 34.7 and 17.3 LMH, respectively, when using deionized water as the FS [78,79]. Conversely, particles coated with hyperbranched polyglycerol have been investigated as the DS using Hydration Tech. Innovations membranes. In this case, the DS concentration range was considerably higher, spanning 300–500 g·L^−1^. However, despite the increased concentration, the resulting water fluxes were lower compared to the previous examples, falling within the range of 3.0–7.2 LMH [80,81,82]. However, given the variations in experimental conditions across these examples and the reviewed literature presented in Table 3, drawing definitive conclusions about the performance of each type of MNPs as a DS remains challenging. 

With regard to the regeneration step of the diluted DS, a number of different methods may be employed, including membrane processes, such as ultrafiltration, and thermal processes, such as heating. Nevertheless, the prevailing approach in the literature for the recovery of MNPs is the application of an external magnetic field. The studies presented in Table 3 clearly illustrate the principal advantage of utilizing MNPs as the DS in FO, namely, their simple recovery by means of an external magnetic field, which results in a cost-effective regeneration step with almost complete recovery of the particles. 

The phenomenon of superparamagnetism is evidenced by the negligible coercivity and remanence, as well as the values of saturated mass magnetization, which fall within the range of 3.8–78 emu g^−1^, as illustrated in Table 3. This property allows the particles to be recovered by an external magnetic field, whereby their magnetic properties are lost when the magnetic field is removed and they are prevented from attracting each other, thus reducing the risk of agglomeration. Even at low values of saturated mass magnetization, around 5 emu g^−1^, the particles can be manipulated by a magnetic field, achieving a recovery rate of 63.4% [15], which serves to highlight their enormous potential. 

There are different alternatives for the recovery of the MNPs from the DS. High gradient magnetic separation (HGMS) columns and open gradient magnetic separation (OGMS) systems are some of the processes used for the recovery of MNPs in large-scale applications. However, these processes have several limitations; they require large and expensive pieces, as well as the high amount of energy required for separation [60]. In contrast, micro magnetic separators (MMS) have emerged as an attractive alternative for small applications. MMS have several advantages, including (i) high efficiency, (ii) portability, (iii) rapid and selective separation, (iv) low cost to produce, (v) high surface area/volume ratio, (vi) reduction of the use of reactants and waste production, (vii) precise control of the fluid flow, and (viii) the capability of continuous separation [21,60]. Additionally, the most important advantage of MMS is the use of permanent magnets as a power-free magnetic field source, as opposed to the energy-intensive HGMS and OGMS [21]. 

In addition to the information shown in Table 3, a number of studies have been conducted to assess the recyclability of MNPs following their utilization as the DS in FO experiments. The water fluxes obtained with the recovered MNPs exhibited a slight decrease in comparison to those of the fresh MNPs. In 2023, Hassanein et al. reported a 25% reduction in water flux after four cycles of MNP reuse, while Shoorangiz et al. observed a 22% reduction after three cycles [14,88]. Nevertheless, other authors have observed a lower water flux reduction, at 11% [63], and even values as low as 3–6% after three cycles [86,91]. These findings demonstrate that the recovered MNPs can be effectively reused in subsequent FO cycles. The reduction in water flux can be attributed to a number of factors, including (i) particle aggregation, (ii) the loss of a small fraction of the MNPs during the recovery process [85], (iii) membrane fouling, and (iv) the interaction between the membrane and MNPs [14,79]. To offset the reduction in water flux when using recovered MNPs, strategies that leverage their magnetic properties can be employed. Examples of such strategies include the use of magnetic composite FO membranes [77] or the implantation of an external magnetic field controller during the FO process in order to reduce the interaction between the membrane and the MNPs [79]. Furthermore, ultrasonication can be employed to reduce the agglomeration of the recovered particles, thereby restoring their performance in the FO process [71].

Despite the considerable potential of utilizing MNPs as a DS, further research is required in order to render them economically and technically viable prior to their implementation as a sustainable DS on a large scale [13,88]. Until now, previous studies have been conducted on a laboratory scale. Consequently, the integration of MNPs into the FO process would be highly advantageous for technological applications in small-scale processes. This approach helps to circumvent technical challenges, including issues such as agglomeration, membrane interaction, and more complex recovery that might arise in larger-scale processes. Furthermore, there may also be economic and technical challenges due to the large amount of nanoparticles required for the process. A number of studies, including those by Zhou et al. in 2015, Ge et al. in 2016, and Yang et al. in 2014, 2015, and 2016, have indicated that high concentrations of particles are necessary for the FO process, with concentrations exceeding 100 g·L^−1^ and up to 600 g·L^−1^ [68,80,81,82,83]. Therefore, it is reasonable to consider the use of MNPs in processes that require a smaller volume of the DS in order to exploit the attractive possibilities of MNPs as the DS, while promoting the economic feasibility of the process. In this context, MNPs could be employed as osmotic agents in osmotic pump-based drug delivery systems, which could represent an intriguing strategy to overcome the limitations of current controlled delivery devices.

## 5. Conceptual Proposal of Systems Based on Osmotic Pumps and MNPs

The widespread use of osmotic pumps in controlled drug delivery demonstrates the immense potential of these systems, as shown in Table 1 and Table 2. Although the main application of MNPs in FO is focused on wastewater treatment and water reclamation [15,58,59], this does not exclude the possibility of integrating MNPs into medical devices. The fact that the integration of both systems has not been considered makes it an attractive area for further research, paving the way to a new multidisciplinary field of research among chemical and biomedical engineering, as well as medicine. 

The integration of MNPs into medical devices could have several advantages. First and foremost, this integrated medical device would incorporate all the benefits of conventional osmotic pumps. This would lead to the design of novel medical devices that achieve controlled and constant drug delivery without the need for batteries or any electronic component [19,29]. As a result, it would reduce the dosing frequency and medical intervention [12,19,36]. Furthermore, the utilization of MNPs as osmotic agents would confer several advantages due to the exceptional properties of MNPs. Studies on the use of MNPs in FO processes have demonstrated the high regeneration of the diluted DS composed of MNPs by an external magnetic field, which would facilitate the re-concentration of DS for subsequent use [56,66,86]. Furthermore, the superparamagnetic properties of the particles can be exploited to achieve even more precise drug release by utilizing materials with magnetic or other stimuli-responsive properties, such as magnetic nanocomposite FO membranes [26], or by implementing a constant magnetic field [79]. Furthermore, in terms of performance, MNPs offer negligible RSF across the membrane [13,86,92], thus avoiding any undesirable interaction between the MNPs and the human body. This technology could also facilitate the simultaneous, controlled delivery of multiple drugs with independent release rates from a single device. Moreover, in the case of designing drug release devices in which the drug shares the same reservoir as the osmotic agents, such as EOP- or CPOP-based devices, regulating the delivery orifice can prevent the loss of MNPs. This approach could help maintain the osmotic gradient, achieving better control over the release and avoiding potential interactions between the osmotic agents and the organism.

In conclusion, the proposed drug delivery systems below have the potential to contribute to the development of novel controlled drug delivery systems with excellent and attractive properties. The absence of studies on this proposal highlights the necessity for further research and development of these promising medical devices. This could potentially lead to the creation of affordable treatments for NCDs, particularly in low-income countries, representing a significant stride towards the achievement of Sustainable Development Goals 3 “Good health and well-being” and 10 “Reduced inequalities” set forth in the 2030 Agenda [5,93].

### 5.1. Wearable Device for Drug Delivery

**Figure 7:**
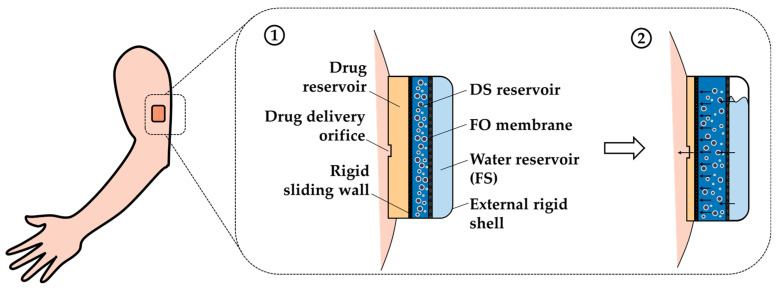
Scheme of an external wearable device for drug delivery based on the integration of osmotic pumps and MNPs: (**1**) wearable device at the initial stage and (**2**) wearable device during drug delivery.

In light of the aforementioned considerations, a wearable drug delivery system based on the integration of osmotic pumps and MNPs is proposed in Figure 7.

The proposed medical device, depicted in Figure 7, comprises the following components: (i) an external rigid shell, (ii) a water reservoir (FS), (iii) an FO membrane, (iv) a DS reservoir, (v) a rigid sliding wall, (vi) a drug delivery orifice, and (vi) a drug reservoir. During the process, as shown in Figure 7(2), the volume of the DS compartment gradually increases due to the water flux generated by difference in osmotic pressure between the FS and DS composed of MNPs. This results in the displacement of the rigid sliding wall, compression of the drug reservoir, and its controlled release. 

It is highly important to carefully select the rigid sliding wall material, as it is directly in contact with the drug. Hence, the ideal material must (i) have enough mechanical strength to avoid undesirable breaks during the process, (ii) be biocompatible, and (iii) be impermeable to prevent interactions with the MNPs and the body. In case of ALZET^®^ osmotic pumps, the material used in the elastic wall is a thermoplastic hydrocarbon elastomer [94]. Due to their excellent properties, these materials are used in several medical applications, including in the fabrication of artificial hearts, mammary implants, and matrices for controlled drug delivery. For instance, thermoplastic elastomers based on poly(styrene-bisobutylene-b-styrene) are used as a coating in drug delivery owing to their (i) low permeability, (ii) elasticity, (iii) thermal stability, and (iv) biocompatibility [95]. Therefore, these kinds of materials might be an appropriate choice for making the rigid sliding wall of the device. 

As the device depicted in Figure 7 is small, the purpose of integrating MNPs with osmotic pumps does not reside in the regeneration of the DS, since it would not have a great impact to regenerate a DS of such a small volume, but in the fact that MNPs have a negligible RSF. As the process is driven by the phenomenon of osmosis, RSF would result in an undesired decrease in the osmotic pressure difference between the FS and DS, leading to a decrease in the flow of water. A reduction in water flow results in a slower increase in the volume of the DS, causing a reduction in the drug release rate as compared to the desired release rate. However, the use of MNPs as the DS would result in better control of the water flux, obtaining a more precise and controlled release in the patient. 

The proposed medical device could potentially replace the disposable mechanical pumps. These disposable drug delivery systems, particularly elastomeric pumps, are receiving special attention over electronic pumps due to their numerous advantages. These include (i) the ease of use, (ii) light weight and small size, (iii) ease of transport, (iv) independence from an external energy source [96,97], (v) disposability, (vi) no programming errors, and (vii) low cost [96]. The continuous infusion rate of elastomeric pumps is achieved through the generation of pressure by an elastomeric balloon. The pressure exerts a force on the drug reservoir, resulting in the infusion of the drug into the patient’s body through a narrow tube without the need for an external energy source [96,98]. This drug delivery system has been successfully employed in a number of fields, including analgesia, chemotherapy, and cardiology [98]. However, these systems have several disadvantages. Firstly, they allow for a faster infusion rate than is prescribed, with rates of 110–150% at the beginning and end of treatment [97]. Secondly, they are dependent on environmental factors, such as temperature, atmospheric pressure, and fluid viscosity variation, as well as height relative to the catheter location [96,97]. Thirdly, they have a lower accuracy compared to electronic pumps. Consequently, the proposed drug delivery system could result in the development of an alternative medical device that overcomes the instability of elastomeric pumps with regard to infusion rates, while maintaining their various advantages. 

### 5.2. Extracorporeal Device for Drug Delivery

**Figure 8:**
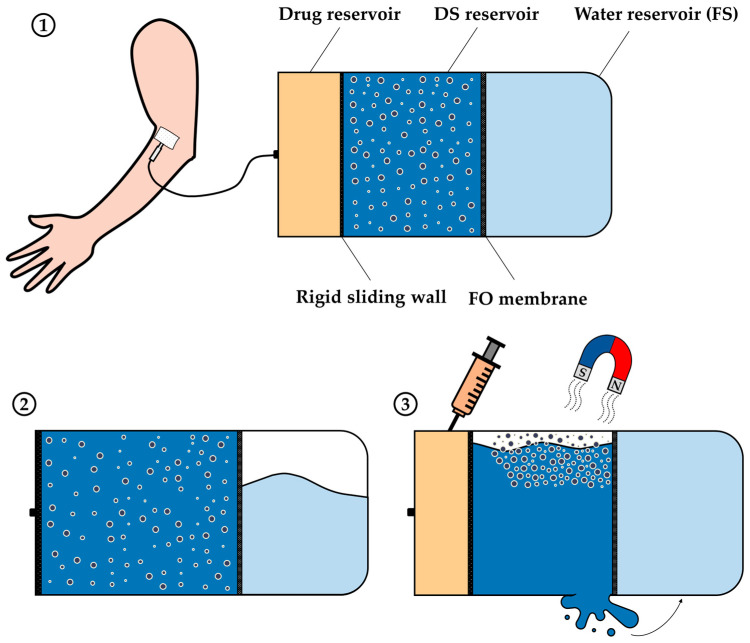
Scheme of an extracorporeal device for drug delivery: (**1**) extracorporeal device at the initial stage, (**2**) extracorporeal device after it has been used, and (**3**) regeneration step of the extracorporeal device.

The combination of MNPs and osmotic pumps could also lead to the development of bigger drug delivery devices, as shown in Figure 8.

The extracorporeal device proposed in Figure 8 is similar to the wearable device in Section 5.1. The water flow from the water reservoir to the DS reservoir across an FO membrane increases the volume of the DS reservoir, moving a sliding wall and causing the release of the drug at a controlled rate. 

Besides the low RSF of the MNPs, the regeneration of the DS in this proposed drug delivery system by an external magnetic field provides an interesting advantage, as MNPs have great potential to be reused in subsequent applications. The proposed design ensures that once the drug contained in the delivery system has been consumed, the water that has migrated from the water into the DS reservoir during the process can be drained using an external magnetic field, as depicted in Figure 8(3). This process allows the MNPs to be reused and the DS to be concentrated to the required level. Once the drug and water have been replenished in their respective reservoirs, the medical drug delivery system can be reused for a new treatment cycle.

Therefore, this drug delivery system represents an innovative alternative to other intravenous administration systems used in hospitals, such as volumetric pumps, syringe pumps, epidural pumps, or other types of infusion pumps [99]. In contrast to conventional infusion pumps, the proposed extracorporeal drug delivery system could also achieve controlled release of the drug but without relying on electricity for its operation, which is particularly valuable in locations with limited access to an electricity supply.

As a summary, Table 4 shows the main advantages that the proposed systems, resulting from the integration of osmotic pumps and MNPs, would have over some of the available drug delivery systems.

### 5.3. Considerations in the Design of Osmotically Driven Drug Delivery Systems

In the design of osmosis-based drug delivery systems, phenomena and parameters that are a possible cause of the reduction in technology efficacy, such as concentration polarization (CP) or reverse solute flux (RSF), must be considered. 

Semipermeable FO membranes are designed to permit the passage of water, while rejecting unwanted compounds. Nevertheless, solute flux is unavoidable [56,59]. In FO, solute diffusion can occur in both directions, where RSF is solute diffusion from the DS side to the FS side. This solute diffusion reduces the osmotic pressure difference, decreasing the water flux across the membrane [56,57]. In the case of drug delivery systems based on osmotic pumps, a decrease in water flux results in a slower drug release with respect to the desired one, which would lead to less precession of the device. However, when using MNPs as osmotic agents, as they have a negligible RSF, this undesired phenomenon could be minimized [13,86,89]. 

**Figure 9:**
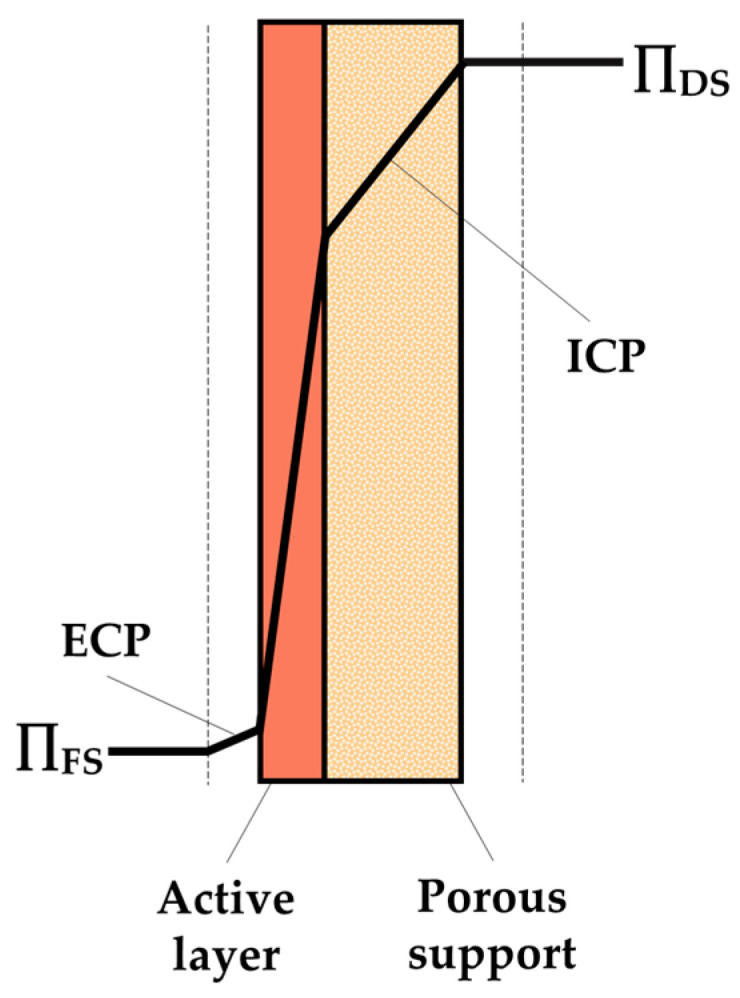
CP in an FO process in an asymmetrical membrane with the FS facing the active layer.

CP, as illustrated in Figure 9, is a phenomenon by which the molecules accumulate on the surface of membranes, resulting in a reduction in the osmotic pressure difference and a subsequent reduction in the water flux. Thus, similar to RSF, a decrease in water flux would result in lower precision in drug release. A distinction can be made between external concentration polarization (ECP), whereby the concentration profile occurs on the external surface of the dense active layer, and internal concentration polarization (ICP), whereby the concentration profile occurs within the porous support of the membrane [17,100].

In dense FO symmetric membranes, ECP can occur on both sides of the membrane. To quantify ECP, the water flux can be expressed as follows [17,100]:

(3) $$J_{w}=A\left[\pi_{DS}\exp\left(-\frac{J_{w}}{k_{DS}}\right)-\pi_{FS}\exp\left(\frac{J_{w}}{k_{FS}}\right)\right]$$

where $k_{DS}$ and $k_{FS}\;\left(m\;s^{-1}\right)$ are the mass transfer coefficients on the DS and FS sides, respectively.

**Figure 10:**
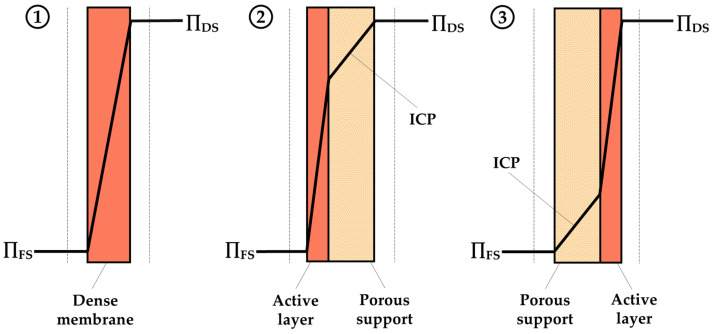
ICP in the proposed drug delivery systems using (**1**) a dense membrane, (**2**) asymmetrical membrane with the FS facing the active layer, and (**3**) asymmetrical membrane with the FS facing the porous support layer.

Since in the proposed drug delivery systems there is no fluid movement, ECP does not occur, and so Equation (3) is simplified as Equation (1), resulting in no decrease in the osmotic pressure difference, as shown in Figure 10(1). 

Conversely, in asymmetrical FO membranes, which are formed by a porous support bounded by a compact active layer, it is essential to consider the ICP phenomenon that occurs within the membrane. Consequently, for asymmetrical membranes, the water flux can be described by considering ECP on the surface of active layer, since it is assumed that no ECP occurs on the support layer side of the membrane, and ICP. When the active layer is in contact with the FS, the water flux is determined by the following equation [17,100]:

(4) $$J_{w}=A\left[\pi_{DS}\exp\left(-J_{w}\;K\right)-\pi_{FS}\exp\left(\frac{J_{w}}{k_{FS}}\right)\right]$$

Considering there is no ECP because there is no fluid movement in the proposed drug delivery systems, as depicted in Figure 10(2), water flux is defined using the following equation:

(5) $$J_{w}=A\left[\pi_{DS}\exp\left(-J_{w}\;K\right)-\pi_{FS}\right]$$

However, if the porous support layer is in contact with the FS, the equation for water flux considering ECP and ICP is described as follows:

(6) $$J_{w}=A\left[\pi_{DS}\exp\left(-\frac{J_{w}}{k_{DS}}\right)-\pi_{FS}\exp\left(J_{w}\;K\right)\right]$$

As there is no ECP in the proposed devices, as illustrated in Figure 10(3), the equation is simplified as follows:

(7) $$J_{w}=A\left[\pi_{DS}-\pi_{FS}\exp\left(J_{w}\;K\right)\right]$$

where $K\left(s\;m^{-1}\right)$ is the solute resistivity inside the porous support layer. It is defined by the following equation:

(8) $$K=\frac{t\;\tau }{D\;\varepsilon }$$

where $D\left(m^{2}\;s^{-1}\right)$ is the diffusion coefficient of the solute in the solution, and t(m), τ(−), and ε(−) are the thickness, tortuosity, and porosity of the support layer of the FO membrane, respectively.

Although ECP is not considered in the proposed devices due to the absence of fluid movement, it is important to note that when designing drug delivery devices based on osmotic pumps with fluid movement, considering ECP could be a key factor in the accuracy of the control system.

In the context of osmotic pumps, CP and RSF are undesirable phenomena that should be considered during the design of the drug delivery system. They lead to a decrease in the osmotic gradient, resulting in reduced water flux across the membrane. This reduction could have a negative impact on the kinetics of drug release. Therefore, understanding the mechanisms underlying this drug release device is crucial. By accounting for all the phenomena that deviate from idealization, more accurate devices can be developed.

Moreover, as the DS dilutes over time, the regeneration of the DS is a critical stage for the viability of the overall FO process. By formulating the DS with MNPs, the diluted DS can be easily regenerated using an external magnetic field after the FO process is completed, as illustrated in Figure 8(3). This regeneration enables the DS to be reused in subsequent cycles.

Recovery using an external magnetic field is attributed to the action of a magnetic force, described by the following equation [60,101,102]:

(9) $$Fm=\mu_{0}\;V_{p}\;M_{p}\nabla {\;H}_{a}$$

where Fm(N) is the magnetic force, $\mu_{0}$ is the permeability of the free space (4 π 10^−7^ H m^−1^), $V_{p}\;(m^{3})$ is the volume of the particle, $M_{p}\;\left(A\;m^{-1}\right)$ is the magnetization of the particle, and $H_{a}\;(T)$ is the applied magnetic field. 

According to Equation (9), the magnetic force is proportional to the volume of the particle, which means that larger volumes result in a greater magnetic force, making separation easier. Nevertheless, size is a critical factor in FO processes with a DS based on MNPs. In order to maintain small particle sizes while generating sufficient magnetic force for MNP recovery, considering the material of the MNPs is critical. The use of MNPs with a high saturation magnetization mass, such as superparamagnetic materials, increases the magnetic force applied by the magnet, as it is proportional to the magnetization of the particle, thus facilitating the separation of the MNPs from the DS [101]. 

Therefore, magnetic recovery of the MNPs from the diluted DS would be a viable option, especially in devices with a higher quantity of MNPs, such as the extracorporeal device proposed. Moreover, MMS could be used for recovery due to several advantages, standing out is the power-free generation of the external magnetic field using a permanent magnet [21,60]. 

### 5.4. Integration in Pharmaceutical Manufacturing

**Figure 11:**
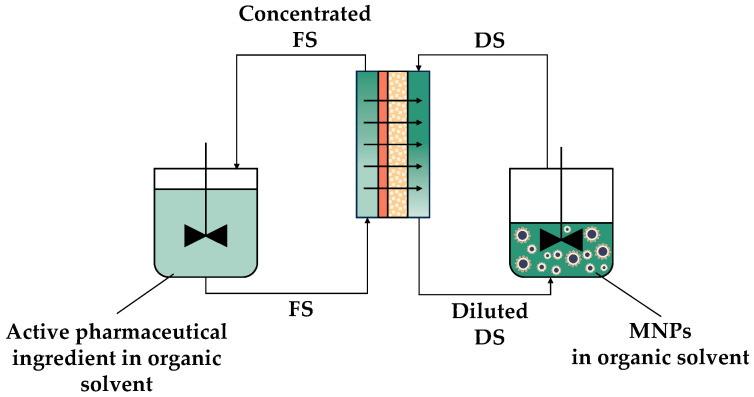
Scheme of an FO process with MNPs as the DS for the concentration of active pharmaceutical ingredients in the pharmaceutical industry.

The integration of technologies also has the potential to be utilized in industries. For instance, it could be employed in the pharmaceutical industry as a concentration or reagent dosing method. In more specific terms, the dissolution of active ingredients in organic solvents is a common procedure in the synthesis of pharmaceutical drugs. This process necessitates the separation and purification of the active ingredients in order to achieve the desired drug quality [103], with this stage representing a significant proportion of the investment in the pharmaceutical industry. However, classical purification methods are energy-intensive and use toxic solvents, which has led to the necessity to develop non-thermal, cost-effective, and sustainable purification methods [103,104]. In this context, organic solvent forward osmosis (OSFO) has been investigated as a promising process for concentrating active pharmaceutical ingredients while extracting organic solvents [103]. DSs such as LiCl [105], polyethylene glycol 400 [104,106], and 1-ethyl-3-methylimidazoliumbis(trifluoromethylsulfonyl)imide [103] have been employed in OSFO, thereby demonstrating the potential of the technology. Nevertheless, the RSF, CP, and the energy-intensive regeneration of the DS represent limitations to the process. As discussed in previous sections, the formulation of DS with functionalized MNPs could achieve the desired concentration of active pharmaceutical ingredients, enabling the regeneration of the DS with a low energy requirement. In this regard, Figure 11 depicts a scheme for integrating MNPs and FO as a concentration method in the pharmaceutical industry. 

**Figure 12:**
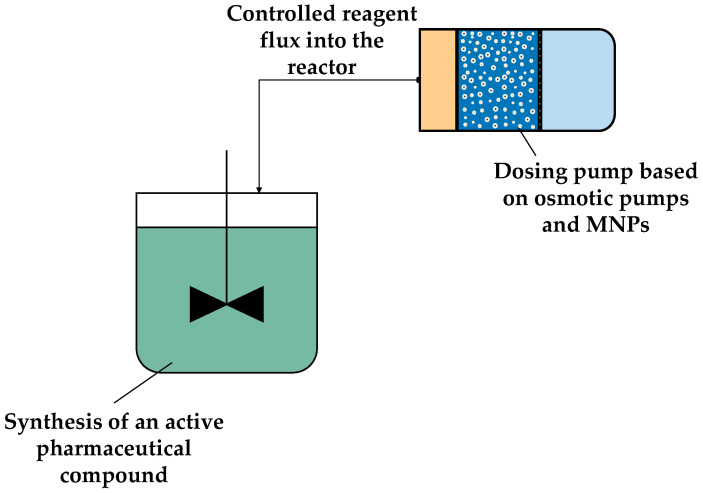
Scheme of the synthesis of an active pharmaceutical compound using a dosing pump based on the integration of osmotic pump and MNPs.

The integration of MNPs and FO could also lead to the development of a novel dosing pump with low energy requirements, as shown in Figure 12. This dosing pump could be implemented in the pharmaceutical industry for a controlled and constant flux of liquid reagent into reactors, based on the same mechanism as the extracorporeal device for drug delivery described in Section 5.2.

The use of MNPs in this type of dosing pumps would allow the regeneration of the DS composed of MNPs by applying an external magnetic field, resulting in a DS regeneration stage with a low energy requirement. This is particularly the case when permanent magnet-based DS regeneration systems, such as MMS, are employed, as they are less energy-intensive compared to traditional regeneration methods [13,20,21]. However, in this context, the application of this type of technology in the pharmaceutical industry is constrained by the scale of the process and the large required amount of MNPs, which currently renders it economically and technically unviable. Large-scale synthesis and functionalization of MNPs remains challenging since conventional methods, such as co-precipitation and thermal decomposition, are batch processes, resulting in a poor control of the variables if large volumes of MNPs are synthesized. Although both methods can be scaled up, co-precipitation requires proper control of process variables such as the temperature, reaction time, and precursors, with the control of particle size and distribution being a bottleneck in this synthesis method. In the case of thermal decomposition, although this method achieves a better control of the size of the particle and a narrow particle size distribution, finding the optimal conditions is challenging. Moreover, thermal decomposition is an expensive and energy-consuming method [61,107]. Consequently, although MNPs hold considerable potential for use in the pharmaceutical industry, the most promising application at present is their integration in osmotic pumps.

## 6. Outlook and Future Perspectives

MNPs have recently gained interest due to their remarkable properties, which have led to their use in a variety of fields. These include photocatalytic processes, wastewater treatment, high-performance separation processes, and drug delivery. In the context of FO, MNPs have been extensively studied for their use as a DS due to their ease of recovery, high functionalization capacity, and insignificant RSF. The application of MNPs in FO is focused on environmental applications, particularly in wastewater treatment and desalination. This review is the first to highlight the significant potential of integrating MNPs and medical devices for drug delivery, a topic that has not been considered in the literature to date. 

The authors are confident that the findings presented in this study represent a significant advancement in the field of drug delivery systems, offering novel insights and potential avenues for enhancing their performance. The exceptional performance of osmotic pumps in drug delivery and MNPs in FO processes provides a rationale for exploring the integration of both technologies and the potential emergence of a novel approach to drug release.

Furthermore, this could represent a social breakthrough in addition to contributing to the generation of knowledge about medical devices for drug delivery. The advancement of these controlled drug delivery systems could result in several benefits, including a reduction in the frequency of drug administration, the elimination of the need for electronic devices, and the possible delivery of multiple drugs simultaneously. Additionally, MNP properties, such as the negligible RSF and superparamagnetism, can improve the precision in drug release and the reusability of the DS. This would be beneficial for the treatment of NCDs in developing countries, where the management of these diseases is a leading cause of death, by providing continuous and cost-effective healthcare.

Consequently, it would be highly advisable to invest economic and research efforts in the study and development of this type of device. Investing in the assessment of their technical feasibility and optimisation is therefore necessary in order to develop affordable devices for the treatment of NCDs.

## Figures and Tables

**Table 1:** Examples of commercial drugs based on osmotic pumps.

Drug	Active Ingredient	Application	Ref.
Actoplus Met XR (Takeda Pharmaceuticals Company, Tokyo, Japan)	Pioglitazone and metformin hydrochloride	Glycaemic control in adults with type 2 diabetes mellitus	[42]
Adalat Oros (Bayer, Leverkusen, Germany)	Nifedipine	Angina and hypertension	[43]
Concerta^®^ (Janssen Pharmaceuticals, Inc., Beerse, Belgium)	Methylphenidate hydrochloride	Attention deficit hyperactivity disorder	[44]
Ditropan XL^®^ (Janssen Pharmaceuticals, Inc., Beerse, Belgium)	Oxybutynin chloride	Overactive bladder	[45]
Elafax^®^ XR (Gador S.A., Bueno Aires, Argentina)	Venlafaxine	Major depressive disorder, generalized anxiety disorder, and panic disorder	[46]
Glucotrol XL (Pfizer, Inc., New York, NY, USA)	Glipizide	Improve glycaemic control in patients with type 2 diabetes mellitus	[47]
Osmolex ER^TM^ (Supernus Pharmaceuticals, Rockville, MD, USA)	Amantadine hydrochloride	Parkinson’s disease and drug-induced extrapyramidal reactions in adults	[48]
Procardia XL^®^ (Pfizer, Inc., New York, NY, USA)	Nifedipine	Angina and hypertension	[49]

**Table 2:** Summary of the most recent studies related to osmotic pumps.

Osmotic System	Osmotic Agent	Administration	Active Ingredient	Target	Ref.
CPOP	Potassium chloride and mannitol	Oral	Paliperidone	-	[34]
	Lactose monohydrate and fructose		Enalapril maleate	-	[9]
EOP	Hydroxypropyl methylcelluloses		Diltiazem hydrochloride	-	[11]
	Sodium chloride		Valganciclovir HCl	Beagle dogs	[12]
PPOP			Diltiazem, ambroxol, paracetamol, etc.	-	[10]
EOP	Mannitol with polyethylene oxide	Implanted on the jugular vein	Fenofibrate-loaded solid lipid coating + LDL antibodies	White pigs	[50]
HTP *	Sodium chloride	Implanted subcutaneously	PDE8 inhibitor	C57BL/6 mice	[39,51]
HTP * Model 1002		Implanted subcutaneously and linked to the ventricle	tcDNA	Mdx52 mice	[41,51]
HTP * Model 1004		Implanted in the right ear	Fluvastatin	CBA/CaJ mice	[38,51]
HTP * Model 2001		Implanted subcutaneously	Meloxicam	Pigeons	[19,51]
HTP * Model 2006			Angiotensin II	Mst1^−/−^ and C57BL/6 wild-type mice	[40,51]
HTP * Model 2006		Implanted in the left ear	Artificial perilymph	Guinea pigs	[51,52]
HTP * Model 2ML4		Implanted subcutaneously in the lumbar area	Isoform FS-288	Sprague–Dawley rats	[51,53]
HTP * Model AP2004		Implanted subcutaneously	Angiotensin II	Apoe^−/−^	[51,54]
HTP * Model 2002		Connected to the neurite outgrowth chamber	Neurotrophin-3	3D-printed neurite outgrowth chamber	[51,55]

* (ALZET^®^, Durect Corporation, Cupertino, CA, USA).

**Table 3:** Summary of studies using MNPs as the DS in the FO process.

Particles and Coating/Functionalization	MNP Synthesis Method	Particle Size (nm)	FO Membrane	Draw Solution	Osmotic Pressure (bars)	Feed Solution	Water Flux (LMH)	Reverse Solute Flux (gMH)	MNP Recovery Method	Saturated Mass Magnetization (emu·g^−1^)	Recovery	Ref.
MNPs coated/functionalized with organic acids and their derivatives												
Citric acid-coated MNPs	Co-precipitation	3–7	AIM™ HFFO membrane (Aquaporin A/S, Kongens Lyngby, Denmark); *A* = 180.0 cm^2^	3.70% (*w*/*w*)	18.7	Deionized water	9.2	0.08	-	44.0	-	[67]
		40	Polyethersulfone thin film composite FO membranes	600.00 g·L^−1^	80.0	3.5% (*w*/*w*) NaCl	8.5	>0.10	Magnetic field and nanofiltration	60.0	≈100.0% *t* = 10 min	[68]
Dehydroascorbic acid-coated MNPs		20	Cellulose triacetate/ cellulose acetate FO membrane; *A* = 40.0 cm^2^	0.06 g·L^−1^	-	Deionized water	6.0	-	Magnetic field	77.7	≈100.0%	[69]
Multicoated MNPs with polyacrylic acid as a terminal hydrophilic ligand		12	AIM™ HFFO (Aquaporin A/S, Kongens Lyngby, Denmark); *A* = 180.0 cm^2^	0.60%	8.9		4.1	-		-	67.6%	[58]
Polyacrylic acid-coated MNPs	Microwave irradiation and co-precipitation	7	AIM™ HFFO module (Aquaporin A/S, Kongens Lyngby, Denmark); *A* = 180.0 cm^2^	0.70%	12.8		8.1	-		19.4	≈100.0%	[56]
	Thermal decomposition	8–30	Cellulose triacetate FO membrane (Hydration Tech. Innovations, Albany, OR, USA); *A* = 20.0 cm^2^	0.08 M	-		13.9	-		-	≈100.0%	[70]
						35 g·L^−1^ NaCl	6.3					
		5	Commercial FO membrane (Hydration Tech. Innovations, Albany, OR, USA); *A* = 8.0 cm^2^	0.08 M	70.9	Deionized water	12.0	-	Ultrafiltration	-	≈100.0%	[71]
						3.5% (*w*/*w*) NaCl	3.0					
		20–30		0.05 M	-	Deionized water	7.7	-	Magnetic field	-	≈100.0%	[72]
Polyethylene glycol dicarboxylic acid- functionalized SiO_2_-coated MNPs		-	FO membrane (Aquaporin A/S, Kongens Lyngby, Denmark); *A* = 31.0 cm^2^	8.00 g·L^−1^	-	40 mg·L^−1^ NaCl	12.2	-		5.0	63.4%	[15]
Poly-sodium acrylate-coated MNPs	Co-precipitation	520	AIM^TM^ membrane (Aquaporin A/S, Kongens Lyngby, Denmark); *A* = 33.2 cm^2^	7.00%	9	Deionized water	3.8	0.05	-	25.0	-	[73]
		77–166	Cellulose triacetate FO membrane; *A* = 98.0 cm^2^	1.00% (*w*/*w*)	1.3		-	-	-	-	-	[74]
Poly-sodium acrylate-coated MNPs	Thermal decomposition	7	Specialized carbon nanotube FO membrane (Porifera Inc., San Leandro, CA, USA); *A* = 42.0 cm^2^	0.07% (*w*/*v*)	25.3	Deionized water	11.7	-	Magnetic field and heating	-	≈100.0% *t* = 1–5 min	[75]
		9		0.13% (*w*/*w*)	11.4		5.3	-	Magnetic field	-	≈100.0% *t* = 5 min	[76]
Sodium oleate-coated MNPs	Co-precipitation	32	Cellulose triacetate magnetic composite FO membrane; *A* = 23.7 cm^2^	0.1 g·L^−1^	-	1.0 M NaCl	11.4	-		-	84.4%	[77]
Tri-sodium citrate- functionalized SiO_2_-coated MNPs		20–40	Cellulose triacetate FO membrane; *A* = 14.0 cm^2^	80.00 g·L^−1^	125.6	Deionized water	17.1	1.50		32.7	≈100.0%	[66]
						0.5 M NaCl	2.7	-				
Tri-sodium citrate-coated MNPs		66–69	Cellulose triacetate FO membrane (Hydration Tech. Innovations, Albany, OR, USA); *A* = 140.0 cm^2^	2.00 g·L^−1^	-	Deionized water	34.7	-	-	-	-	[78]
		3–8	Cellulose triacetate FO membrane (Hydration Tech. Innovations, Albany, OR, USA); *A* = 20.0 cm^2^	0.02 g·L^−1^	-		17.3	-	-	-	-	[79]
MNPs coated/functionalized with organic polymers												
Chitosan-coated MNPs	Co-precipitation	20	Cellulose triacetate/ cellulose acetate FO membrane; *A* = 40.0 cm^2^	0.06 g·L^−1^	-	Deionized water	5.0	-	Magnetic field	70.3	≈100.0%	[69]
Hyperbranched polyglycerol carboxylate-coated MNPs	Thermal decomposition	29	OsMem™ (Hydration Tech. Innovations, Albany, OR, USA); *A* = 50.0 cm^2^	500.00 g·L^−1^	15.8		7.2	-	Ultrafiltration	18.7	≈100.0%	[80]
Hyperbranched polyglycerol-coated MNPs		21		300.00 g·L^−1^	15.2		6.2	-	-	20.7	-	[81]
Hyperbranched polyglycerol-coated MNPs functionalized with succinic anhydride moieties		24	OsMem™ (Hydration Tech. Innovations, Albany, OR, USA); *A* = 2.4 cm^2^	400.00 g·L^−1^	9.7		3.0	-	Ultrafiltration	19.3	≈100.0%	[82]
Magnetic poly (N-isopropylacrylamide-co-sodium 2-acrylamido-2-methylpropane sulfonate) nanogels	Co-precipitation	271	Cellulose triacetate with an embedded polyester screen mesh FO membrane (Hydration Tech. Innovations, Albany, OR, USA); *A* = 23.0 cm^2^	100.00 g·L^−1^	3.3		0.6	-	Magnetic field and heating	25.3	≈100.0% *t* = 20 min	[83]
Poly (N-isopropylacrylamide)-coated MNPs	Thermal decomposition	7	Specialized carbon nanotube FO membrane (Porifera Inc., San Leandro, CA, USA); *A* = 42.0 cm^2^	0.07% (*w*/*v*)	25.3		11.7	-		-	≈100.0% *t* = 1–5 min	[75]
Polyethylene glycol 4000- coated MNPs	Co-precipitation	-	Cellulose triacetate FO membrane (Fluid Tech. Solutions, Inc., San José, CA, USA); *A* = 49.0 cm^2^	10.00 g·L^−1^	-	Deionized water	14.9	-	Magnetic field	-	≈100.0% *t* = 2 min	[65]
Polyethylene glycol-coated MNPs	Polyol process	9–32	Cellulose triacetate FO membrane (Hydration Tech. Innovations, Albany, OR, USA); *A* = 20.0 cm^2^	0.08 M	-		11.3	-		-	≈100.0%	[70]
						35 g·L^−1^ NaCl	5.2					
Polyethylene glycol dicarboxylic -coated MNPs	Thermal decomposition	13	Flat sheet FO membrane (Hydration Tech. Innovations, Albany, OR, USA); *A* = 12.0 cm^2^	0.07 M	73.9	Deionized water	9.1	-		35.5	≈100.0%	[84]
Poly(amidoamine) dendrimer-coated MNPs	Co-precipitation	17	Thin film composite FO membrane (Porifera Inc., San Leandro, CA, USA); *A* = 42.0 cm^2^	30.00 g·L^−1^	-		12.9	-		48.0	100.0% *t* = 2 min	[14]
Poly(sodium styrene-4-sulfonate)-co-poly (N-isopropylacrylamide)-coated MNPs	Thermal decomposition	5	Thin film composite FO membrane (Hydration Tech. Innovations, Albany, OR, USA)	33.00% (*w*/*w*)	55.7	Deionized water	14.9	-	Magnetic field, ultrafiltration, and heating	11.1	≈100.0%	[85]
						3.5% (*w*/*w*) NaCl	2.7					
Sodium alginate sulfate-functionalized SiO_2_-coated MNPs	Co-precipitation	63–76	Cellulose triacetate *A* = 14.0 cm^2^	60.00 g·L^−1^	118.8	Deionized water	8.5	0.23	Magnetic field	50.6	100.0%	[86]
Triethylene glycol-coated MNPS	Thermal decomposition	20	Commercially FO membrane (Hydration Tech. Innovations, Albany, OR, USA); *A* = 8.0 cm^2^	0.20 M	-		6.0	-	-	20.0	-	[71]
MNPs coated/functionalized with polysaccharides												
Dextran-coated MNPs	Co-precipitation	10	Commercially FO membrane (Hydration Tech. Innovations, Albany, OR, USA); *A* = 48.0 cm^2^	0.50 M	-	Deionized water	4.0	-	Magnetic field	32.4	≈100.0% *t* = 10–15 min	[87]
						2 g·L^−1^ MgSO_4_	3.0					
D-Xylose-coated MNPs	Hydrothermal method	-	Commercial FO membrane (Hydration Tech. Innovations, Albany, OR, USA); *A* = 1.8 cm^2^	6.50% (*w*/*v*)	1.5	Deionized water	2.9	-		30.0	≈100.0%	[88]
						0.01 M NaCl	1.3					
Pectin-coated MNPs	Co-precipitation	390	Polyamide FO membrane (Porifera Inc., San Leandro, CA, USA); *A* = 12.6 cm^2^	0.50%	-	Deionized water	26.6	-		18.6	≈100.0% *t* = 12–16 min	[89]
						1% (*w*/*w*) NaCl	6.6					
MNPs coated/functionalized with other organic compounds												
3-(Trimethoxysilyl) propyl methacrylate-functionalized SiO_2_-coated MNPs	Co-precipitation and sol-gel method	80	Thin film composite FO membrane; *A* = 4.9 cm^2^	-	-	Deionized water	10.2	-	Magnetic field	44.2	≈100.0%	[90]
Poly (deep eutectic solvent)-coated MNPs	Solvothermal procedure	15–25	Cellulose triacetate FO membrane (Hydration Tech. Innovations, Albany, OR, USA); *A* = 15.0 cm^2^	3.50 g·L^−1^	68.9		17.9	0.12		60.4	≈100.0%	[91]
Bare MNPs and MNPs coated/functionalized with inorganic compounds												
Bare MNPs	Co-precipitation	10–20	FTSH2O (Porifera Inc., San Leandro, CA, USA); *A* = 42.0 cm^2^	-	-	-	1.9	-	-	-	-	[16]
		127	Polyamide FO membrane (Porifera Inc., San Leandro, CA, USA); *A* = 12.6 cm^2^	5.00% (*w*/*w*)	-	Deionized water	35.7	-	Magnetic field	3.8	≈100.0% *t* = 7 min	[92]
						20 g·L^−1^ NaCl	2.5					
EDTA-functionalized SiO_2_-coated MNPs	Hydrothermal method	280	Polyamide thin film composite FO membrane (Porifera Inc., San Leandro, CA, USA); *A* = 20.0 cm^2^	60.00 g·L^−1^	-	0.5 g·L^−1^ octanoic acid	9.6	-		18.7	>90.0%	[59]
Potassium-functionalized iron oxide-doped carbon nanofiber MNPs	Co-precipitation	4500	FTSH2O™ (Sterlitech Corporation, Auburn, WA, USA); *A* = 42.0 cm^2^	0.10% (*w*/*v*)	86.1	Deionized water	3.4	0.10	-	22.3	-	[20]
						1.0 M NaCl	2.1					
SiO_2_-coated MNPs	Thermal decomposition	-	FO membrane (Aquaporin A/S, Kongens Lyngby, Denmark); *A* = 31.0 cm^2^	8.00 g·L^−1^	-	40 mg·L^−1^ NaCl	11.0	-	Magnetic field	5.0	83.9%	[15]

**Table 4:** Summary of the main differences between conventional drug delivery methods and the proposed drug delivery systems.

	Disposable Mechanical Pumps	Intravenous Administration Systems	Wearable Device	Extracorporeal Device
Type of system	Existing		Proposed	
Transport	×	×	✓	×
Reusability	×	✓	✓	✓
Independence of an energy source	✓	×	✓	✓
Independence of environmental factors	×	✓	✓	✓

## Data Availability

No new data were created or analysed in this study. Data sharing is not applicable to this article.

## Abbreviations

CP	Concentration polarization
CPOP	Controlled porosity osmotic pump
DS	Draw solution
ECP	External concentration polarization
EOP	Elementary osmotic pump
FO	Forward osmosis
FS	Feed solution
gMH	g m^−2^ h^−1^
HGMS	High gradient magnetic separation
HLP	Higuchi–Leeper pump
HTP	Higuchi–Theeuwes pump
ICP	Internal concentration polarization
LMH	L m^−2^ h^−1^
MD	Membrane distillation
MMS	Micro magnetic separator
MNP	Magnetic nanoparticle
NCD	Noncommunicable or chronic disease
NP	Nanoparticle
OGMS	Open gradient magnetic separation
OSFO	Organic solvent forward osmosis
PPOP	Push–pull osmotic pump
RNP	Rose–Nelson pump
RO	Reverse osmosis
RSF	Reverse solute flux
UF	Ultrafiltration
